# Pain characteristics among individuals with Duchenne muscular dystrophy according to their clinical stage

**DOI:** 10.1186/s12891-022-05504-5

**Published:** 2022-06-04

**Authors:** Aram Kim, Mina Park, Hyung-Ik Shin

**Affiliations:** 1 Department of Rehabilitation Medicine, Myongji Hospital, 14-55 Hwajung-ro, Deokyang-gu, Goyang-si, Gyeonggi-do 412-270 Republic of Korea; 2 Department of Rehabilitation Medicine, Uijeongbu St. Mary’s Hospital, College of Medicine, The Catholic University of Korea, 271 Cheonbo-ro, Uijeongbu-si, Gyeonggi-do 11765 Republic of Korea; 3 Department of Rehabilitation Medicine, College of Medicine, Seoul National University, 101 DaehakRo, Jongno-Gu, Seoul, 03080 Republic of Korea

**Keywords:** Duchenne muscular dystrophy, Pain, Prevalence, Pain interference, Neuromuscular disease

## Abstract

**Background:**

Assessment of pain is not routine, standardized, or well-understood in individuals with Duchenne muscular dystrophy (DMD), even though pain is a common problem reported by more than half of the patients with DMD. Previous studies in this area included multiple neuromuscular diseases with highly variable phenotypes. Therefore, our aim was to specifically focus on DMD and evaluate the comprehensive pain characteristics according to the disease stages, from ambulatory to late non-ambulatory.

**Methods:**

This cross-sectional study was conducted in an out-patient pediatric rehabilitation clinic including 148 male participants with confirmed DMD (14.5 ± 5.3 years of age). Face-to-face interviews were conducted using a structured questionnaire concerning the pain frequency, duration, intensity, location, aggravating/relieving factors, pain interference (Brief Pain Inventory), pain phenotype (PainDETECT Questionnaire), and functional ability (DMD Functional Ability Self-Assessment Tool). Pain characteristics were analyzed according to the clinical stage: ambulatory (Amb), early non-ambulatory (ENA), and late non-ambulatory (LNA).

**Results:**

Of the 148 participants who completed the assessment, 66 (44.6%) reported pain during the previous 4 weeks. There were no differences in the pain duration or intensity among the three groups. Pain location (Amb: calf, ENA: knee, LNA: lumbosacral region), aggravating factor (Amb: ambulation, ENA: transfer, LNA: sitting), and relieving factor (Amb: rest and massage, ENA and LNA: positional change) differed according to the clinical stage. Individuals in the LNA stage reported an increase in the frequency of pain and number of pain sites. The effect of pain on mood was also found to be greater in the LNA group than in the other clinical stages.

**Conclusion:**

There is a change in the pain characteristics, including the location, aggravating/relieving factors, pain frequency, and pain interference, with the progress of the disease in patients with DMD. Thus, clinicians could more efficiently and critically assess and manage the patients’ pain based on these findings.

**Supplementary Information:**

The online version contains supplementary material available at 10.1186/s12891-022-05504-5.

## Background

Duchenne muscular dystrophy (DMD) is a progressive X-linked recessive disorder that causes skeletal muscle weakness with involvement of the cardiac and respiratory muscles in the later stages of disease progression [1]. Patients born after 1990 have a median life expectancy of 28.1 years (95% CI 25.1, 30.3) [2]. Although currently no cure exists for DMD, comprehensive multidisciplinary care, including assisted ventilation as needed, can prolong the survival, with some individuals surviving into their 40s [3–5]. Improving the quality of life (QOL) is an essential goal of the medical care in patients with DMD. In previous studies, pain was associated with the physical health domain of QOL in adults patients with DMD, and several areas of daily life (leisure activity, ability to move, and mood) were affected by pain in patients with muscular dystrophy [6–9].

Pain is a common aspect of DMD, reported by more than half of the individuals with DMD [6, 10, 11]. Pangalia et al. [7] and Zebracki et al. [12] reported a prevalence of pain of 73.4 and 54%, respectively, among individuals with DMD, with a pain intensity of 2.59 ± 1.21 and 1.73 ± 1.58 measured on a 10-point numeric rating scale (NRS), respectively. The common sites of pain were the legs, pelvic region, and back [7, 12]. However, the pain characteristics in DMD have not been evaluated according to the clinical stages of the disease, namely the ambulatory (Amb), early non-ambulatory (ENA), and late non-ambulatory (LNA) stages. Knowledge of the characteristics of pain at the different clinical stages could be the first step in facilitating the development of an effective management strategy for each patient group. Accordingly, our study aimed to conduct a comprehensive evaluation of pain characteristics (frequency, duration, intensity, location, pain phenotype, aggravating/relieving factors, and pain interference on daily activity) among individuals with DMD, according to the different clinical stages of the disease.

## Methods

### Participants

This cross-sectional study included male participants with a diagnosis of DMD who visited our pediatric rehabilitation clinic between April 2020 and February 2021. The diagnosis of DMD was confirmed by genetic testing, including multiplex polymerase chain reaction and direct sequencing to detect mutations of the dystrophin gene. Other inclusion criteria were age ≥ 7 years and no significant cognitive impairment at the discretion of the rehabilitation physician.

Written informed consent forms were signed after the participants understood the purpose and content of this study. If the participant was under than 19 years old, both written informed consent were obtained from their legal guardians and written assent from the participants were obtained. A total of 150 individuals with DMD were approached for this study, and 148 of them agreed to participate. The reason given for not agreeing to participate in the study was “lack of time.” The participants were interviewed in the clinic and completed a structured questionnaire on pain and related factors. If the study participants were unable to understand questionnaire items, one of the authors would explain the item to help them complete the questionnaire. The questionnaire required approximately 20 min to complete for those with pain and 10 min for those without pain. This study was approved by our Institutional Review Board (IRB No. 2004–193-1119).

### Measures

Age, surgical history, presence of scoliosis, and use of non-invasive ventilation were obtained from the medical records. Scoliosis was confirmed through an x-ray, and Cobb angle of more than 10° was considered significant [5, 13]. One of the authors, AK, measured the major joint (hip, knee, ankle, shoulder, elbow, and wrist joint) range of motion using a goniometer according to the American Medical Association guides [14] and recorded the presence of contracture (limitation of the range of motion) by location. The ENA group was defined as a non-ambulatory state in those < 15 years of age; the LNA group was defined as a non-ambulatory state in those ≥15 years of age [15, 16].

### Pain frequency, duration, and intensity

Pain frequency was rated using a 7-point Likert scale, with anchors at “1,” none of the time, and “7,” all day, as previously described [17]. Pain duration was measured using a 4-point Likert scale [18], as follows: “1,” < 1 h; “2,” a few hours; “3,” half of the day; and “4,” all day. Pain intensity was measured using a 11-point NRS, with anchors at “0,” no pain, and “10,” pain as bad as could be. The worst and average pain intensity were the numbers that best described their pain at its worst and on average during the past 4 weeks, respectively. The NRS has been reported to be reliable and valid for children as young as 5 years of age [11]. The location of pain was marked using a body map for children, as per Savedra et al. [19, 20], and was coded as follows: head, chest-abdomen, spine (cervical, thoracic, lumbosacral), upper extremities (shoulder, elbow, wrist-hand), and lower extremities (buttock, hip, thigh, knee, calf, ankle-foot).

### Aggravating and relieving factors

The participants were asked to freely dictate or write down the aggravating/relieving factors for pain and were allowed to list more than one factor. We provided some examples of relieving factors, such as rest, positional change, and massage, and of aggravating factors, such as sitting, excessive activity, and transfer activity.

### Pain interference

Pain interference was assessed using items from the Korean version of the Brief Pain Inventory (BPI) [21]. The amount of pain interference in the past week was rated using a 11-point NRS, with anchors as “0,” does not interfere, to “10,” interferes completely. We assessed the following domains of the BPI: general activity, sleep, social activity, and mood; working ability and enjoyment of life were not included.

### Pain phenotype

The Korean version of the PainDETECT Questionnaire (KPD-Q) was used for screening the neuropathic component of pain [22, 23]. The KPD-Q is a self-administered questionnaire consisting of four sections: pain intensity, pain gradation (seven questions addressing the quality of the neuropathic pain), pain pattern, and pain radiation. The cut-off values were as follows: score ≤ 12, a neuropathic component is unlikely; 13 ≤ score ≤ 18, uncertain; score ≥ 19, a neuropathic component is likely [22].

### Functional ability

The DMD Functional Ability Self-Assessment Tool (DMDSAT) was used to assess the functional ability. The DMDSAT consists of 24 items across 4 domains of function: arm function, mobility, transfers, and ventilation status. The DMDSAT has good reliability and validity across the entire clinical profile of DMD [15]. The total scale score of the DMDSAT ranges between “0” and “23,” with higher scores indicative of higher functional activity.

### Statistical analysis

All the analyses were performed using SPSS (version 25.0 for Windows, IBM Corp., Armonk, NY, USA). Descriptive statistics were used to report the characteristics of the study sample and pain symptoms. Non-parametric Kruskal–Wallis and Fisher tests were used to analyze the differences between the clinical stages, since Amb, ENA, and LNA groups did not meet the assumption of the normality. Bonferroni correction was used for the post-hoc analysis. The correlation between pain interference and pain intensity was assessed using Spearman’s correlation. The level of significance was set at *p* < 0.05 for all the analyses.

## Results

### Clinical characteristics

The study sample included 148 participants, with a mean age of 14.5 ± 5.3 years. The characteristics of our study sample are summarized in Table 1. Most participants had a contracture at least at one site. Participants with advanced DMD were more likely to have scoliosis and a history of spinal surgery, as well as a lower DMDSAT score: Amb, 20.38 ± 2.96; ENA, 8.39 ± 2.14; and LNA, 5.73 ± 2.96. A non-invasive ventilator was used by 16/51 (31.4%) of the participants in the LNA group.

**Table 1:** Demographic and clinical characteristics of the participants

		Ambulatory, *N* = 62	Early non-ambulatory, *N* = 35	Late non-ambulatory, *N* = 51	Total, *N* = 148
Age (years)^a*^		10.73 ± 3.19	12.86 ± 1.63	20.33 ± 3.68	14.54 ± 5.28
Contracture^b,c^	Total^*^	43 (69.4%)	34 (97.1%)	49 (96.1%)	126 (85.1%)
	Ankle^*^	42 (67.7%)	32 (91.4%)	47 (92.2%)	121 (81.8%)
	Knee^*^	2 (3.2%)	21 (60.0%)	39 (76.5%)	62 (41.9%)
	Hip^*^	1 (1.6%)	1 (2.9%)	8 (15.7%)	10 (6.8%)
	Shoulder	0	0	2 (3.9%)	2 (1.4%)
	Elbow^*^	0	0	6 (11.8%)	6 (4.1%)
	Wrist^*^	0	0	13 (25.5%)	13 (8.8%)
Scoliosis^b*^		0	9 (25.7%)	29 (56.9%)	38 (25.7%)
Spinal surgery^b*^		0	0	10 (19.6%)	10 (6.8%)

^a^Mean ± SD

^b^n (%)

^c^If there were two or more joint contractures in one participant, each was counted separately

^*^Significant difference between the groups (*P* < 0.05)

### Pain

#### Prevalence of pain

Overall, 66/148 (45%) of the participants reported having had pain during the previous 4 weeks. There were no significant differences in the prevalence among the three groups (*p* = 0.386): Amb (24/62; 38.7%), ENA (17/35; 48.6%), and LNA (25/51; 49%). The prevalences were not different according to the presence of scoliosis (34/62, 54.8% with scoliosis and 32/86, 37.2% without scoliosis. There was also no difference in the pain prevalence according to the joint contracture (60/126; 47.6% with contracture and 6/22; 27.3% without contracture).

#### Pain frequency, duration, and intensity

**Fig. 1:**
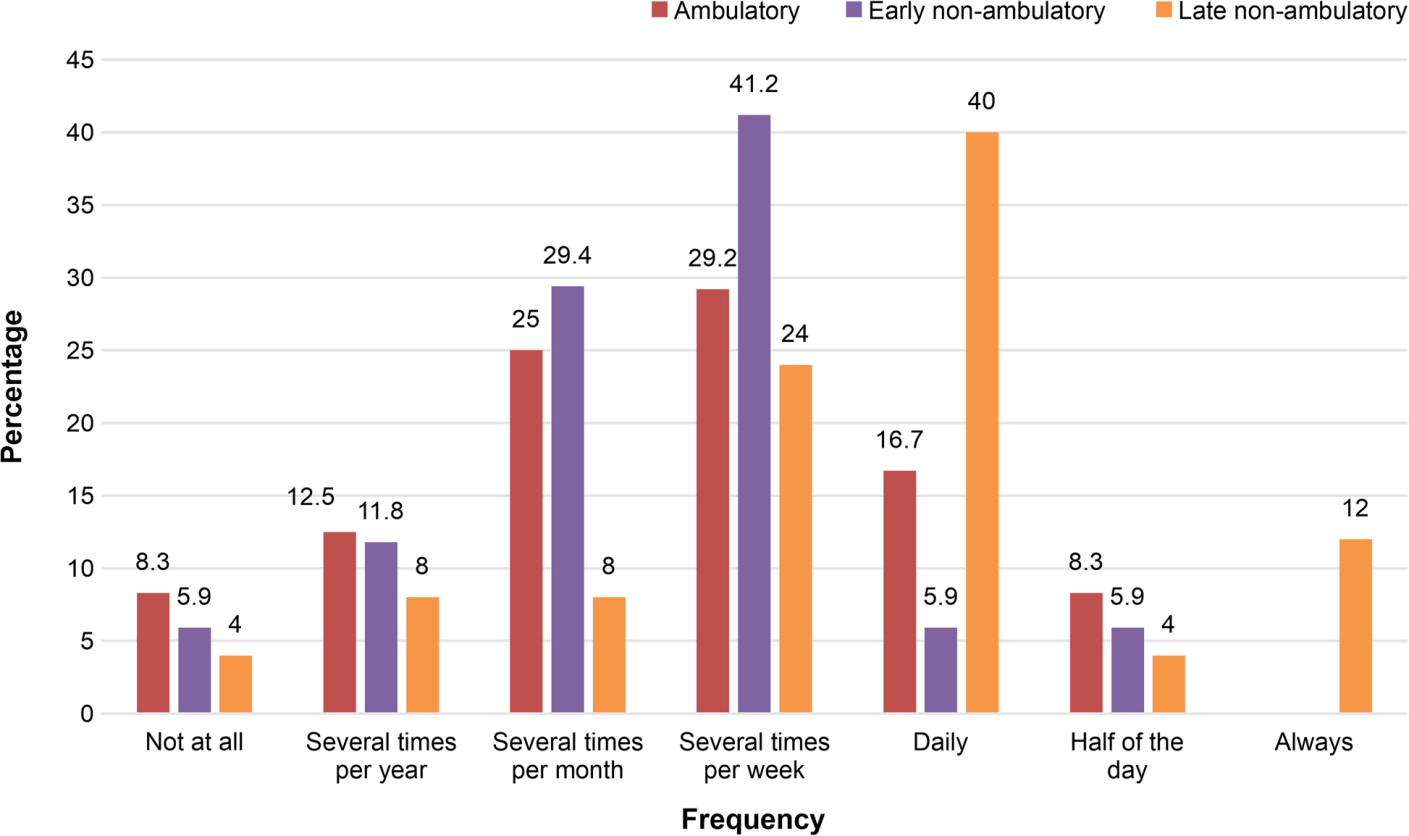
Pain frequency according to the clinical stage among the 66 participants with pain

The most commonly reported pain frequency was “several times per week” in the Amb and ENA groups, and “daily” in the LNA group. The frequency among the three groups were different (*p* = 0.023) and a post-hoc analysis indicated that the LNA group reported a higher frequency of pain compared to both the Amb (*p* = 0.015) and ENA (*p* = 0.006) groups (Fig. 1). There were no differences in the pain duration or intensity among the three groups. More than half of the participants across all groups reported a pain duration of less than 1 h. The worst and average pain intensity during the previous 4 weeks for all the participants were 4.89 ± 2.04 and 3.47 ± 1.87 on the 11-point NRS, respectively, corresponding to a moderate pain intensity. The LNA group had a greater number of pain sites compared to both the Amb and ENA groups (*p* = 0.042; Table 2).

**Table 2:** Pain intensity, duration, and number of pain sites for the 66 participants with pain

		Ambulatory, *N* = 24	Early non-ambulatory, *N* = 17	Late non-ambulatory, *N* = 25	Total, *N* = 66	*p*-value
Intensity	Worst pain	4.83 ± 2.14	4.06 ± 1.56	5.52 ± 2.08	4.89 ± 2.04	0.059
	Average pain	3.33 ± 2.18	2.82 ± 1.43	4.04 ± 1.70	3.47 ± 1.87	0.112
Duration	Less than 1 hour	16 (72.7%)	13 (81.3%)	13 (56.5%)	42 (68.9%)	0.371
	A few hours	2 (9.1%)	3 (18.8%)	7 (30.4%)	12 (19.7%)	
	Half of the day	2 (9.1%)	0	2 (8.7%)	4 (6.6%)	
	All day	2 (9.1%)	0	1 (4.3%)	3 (4.9%)	
Number of pain sites*		1.46 ± 0.72	1.47 ± 0.94	2.24 ± 1.51	1.76 ± 1.18	0.042

^*^Significant difference between the groups (*p* < 0.05)

#### Pain location

**Fig. 2:**
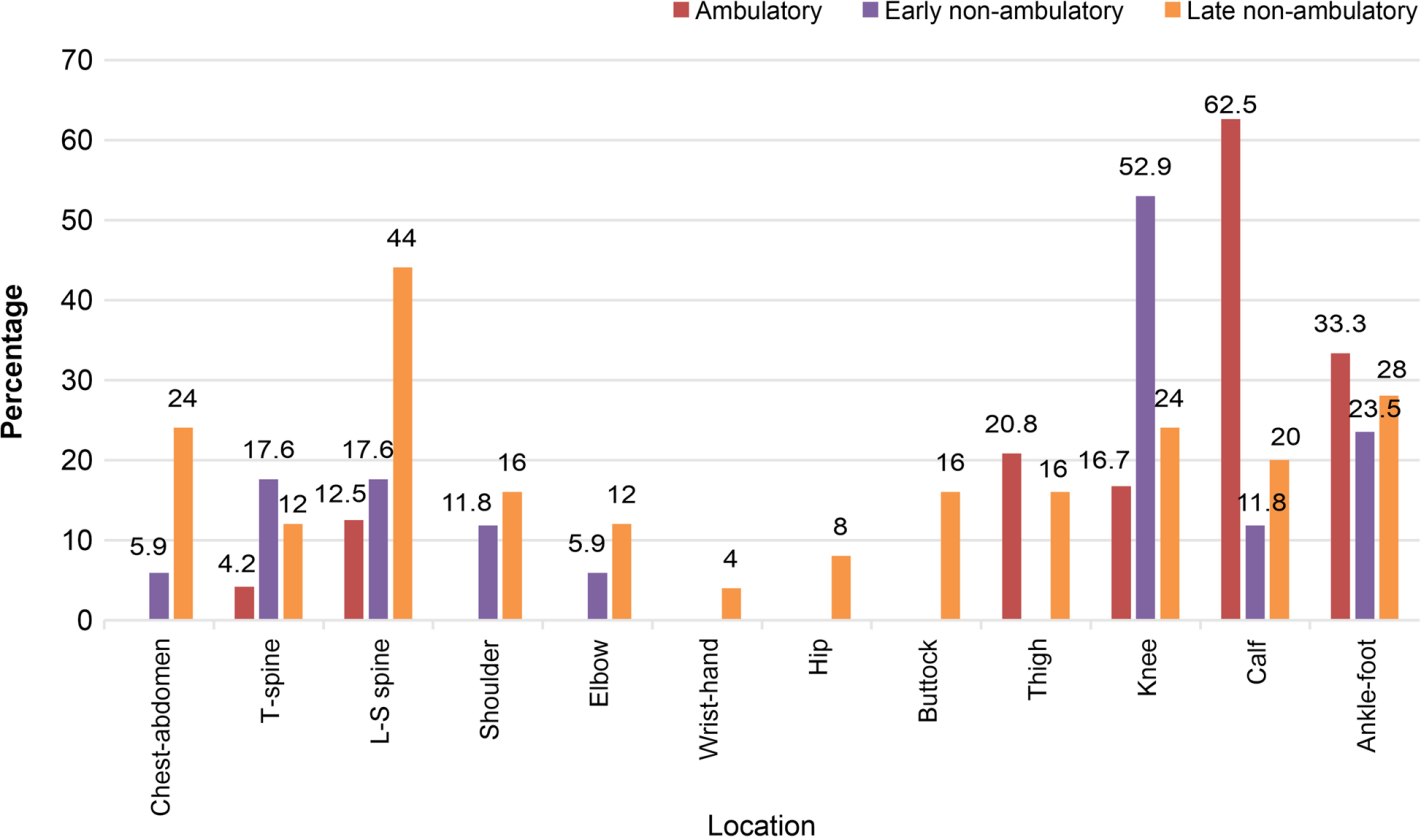
Pain location according to the clinical stage among the 66 participants with pain

The Amb group reported calf pain most frequently (Amb, 62.5%; ENA, 11.8%; LNA, 20%; *p* = 0.001). In contrast, participants in the LNA group reported pain in the lumbosacral region (Amb: 12.5%; ENA: 17.6%, LNA: 44.0%; *P* = 0.04), chest-abdomen (Amb: 0%; ENA: 5.9%; LNA: 24%; *P* = 0.013), and buttocks (Amb: 0%, ENA: 0%, LNA: 16%; *P* = 0.04) more frequently than the Amb and ENA groups (Fig. 2).

#### Aggravating and relieving factors for pain

The pain aggravating and relieving factors are reported in Supplementary Table 1. Among 66 patients with pain, 49 and 50 participants responded to the question regarding aggravating (Amb: 17, ENA: 11, LNA: 21) and relieving factors (Amb: 17, ENA: 13, LNA: 20), respectively. The most common aggravating factors were “ambulation” (14/17), “transfer activity” (5/11), and “sitting” (13/21) in the Amb, ENA, and LNA groups, respectively. The most common relieving factors for pain were “resting” (5/17) and “massage” (5/17) in the Amb group and “positional change” in the ENA (6/13) and LNA (9/20) groups.

#### Pain interference

Among the pain interference items, only general activity items correlated with both the worst and average pain intensity (correlation coefficient, 0.465 and 0.419, respectively; *p* < 0.001). Mood (correlation coefficient, 0.176 and 0.119), social activity (correlation coefficient, 0.120 and 0.179), and sleep (correlation coefficient, 0.171 and 0.191) items did not correlate with the pain intensity. Mood was more affected by pain in the LNA group than in the other groups (*p* = 0.046; Table 3).

**Table 3:** Pain interference for the 66 participants with pain

	Ambulatory, *N* = 24	Early non-ambulatory, *N* = 17	Late non-ambulatory, *N* = 25	Total, *N* = 66	*p-*value
General activity (0–10)	1.79 ± 1.67	2.35 ± 2.42	3.40 ± 2.72	2.55 ± 2.38	0.09
Mood* (0–10)	1.25 ± 2.11	1.94 ± 2.44	2.76 ± 2.49	2.00 ± 2.40	0.046
Social activity (0–10)	0.50 ± 0.98	0.94 ± 2.16	1.80 ± 2.81	1.11 ± 2.18	0.28
Sleep (0–10)	1.08 ± 2.15	1.12 ± 1.62	2.84 ± 3.54	1.76 ± 2.76	0.12

^*^Significant difference between the groups (*p* < 0.05)

#### Pain phenotype

All except three patients reported a KPD-Q score < 12, which corresponded to “neuropathic component is unlikely.” Two participants in the ENA group reported KPD-Q scores between 13 and 18, corresponding to “uncertain” pain characteristics. A 14-year-old boy in the ENA group reported a score > 19, corresponding to “neuropathic pain component is likely.” He experienced pain of moderate intensity several times a month at three body sites (knees, thoracic spine, and lumbosacral spine) which he described as “burning,” “tingling,” and “like an electric shock.”

## Discussion

The prevalence, intensity, and duration of pain in patients with DMD did not differ between the Amb, ENA, and LNA groups. These results support the need for pain assessment and intervention from the ambulatory phase [9, 11, 12]. Additionally, the results of this study suggest an interventional strategy that matches the characteristics of each stage since the pain location and aggravating/relieving factors differed according to the stage of the disease. Meanwhile, we also identified a higher frequency of pain and a greater number of pain sites in the individuals in the LNA stage than in the Amb and ENA stages, and the interference of pain on mood was greater in the LNA group than in the other groups. These results suggest that the pain assessment and interventions are of particular clinical value in the LNA group.

The prevalence of pain in our study sample was 44.6% (66/148 participants), which is lower than the previously reported prevalence of 54 to 73.4% [6, 9, 11]. Tiffreau et al. [7] reported that 73% of the participants with neuromuscular disease reported pain and 62% reported chronic pain, and their mean pain intensity was 6.1/10 with 40% reporting severe pain (a score of ≥7). Hunt et al. [8] reported that two-thirds of young men with DMD suffered from significant daily pain. Previous studies on pain in patients with neuromuscular disease included several motor neuron diseases other than DMD, such as spinal muscular atrophy and Becker muscular dystrophy [9, 11], or reported pain prevalence only for individuals with DMD ≥20 years of age [6], or included a small number of participants [8]. This difference in the study population could be related to the difference in the pain prevalence.

To the best of our knowledge, only one previous study, by Lager et al. [9] has evaluated the pain according to the ambulatory and non-ambulatory clinical stages of progressive neuromuscular disease. The most frequently reported pain sites were the “neck and back” in the non-ambulatory group, compared to the “legs” in the ambulatory group, a finding which is similar to that of our study. They reported that the prevalence, intensity, and frequency of pain did not differ between the two groups, with a reported frequency of pain of “a few times a week.” An increase in the frequency of pain in the non-ambulatory stage might not have been detected in their study owing to the small sample size (55 participants), with mixed disease entities, including muscular dystrophy and spinal muscular atrophy.

In the assessment of pain phenotype in our study among patients with DMD, their pain phenotypes were classified as nociceptive pain (resulting from activation of nociceptors innervating ligaments, small joints, muscle, and tendon) rather than neuropathic pain (resulting from a lesion or dysfunction of the peripheral or central nervous system) [22]. This finding underlines the fact that the pain in patients with DMD could be mainly related to the musculoskeletal conditions and this would emphasize that the pain characteristics need to be identified according the clinical stages since musculoskeletal impairments vary according to the clinical stage of the disease: ankle plantar flexion contracture tends to begin in the Amb stage, hip and knee joint contractures tend to occur in the non-ambulatory stage [24, 25], and scoliosis also generally develops in the non-ambulatory stage [5].

Among the participants in the Amb group in our study, calf pain was the typical pain reported, being aggravated by “standing, walking, and running” and relieved by “rest.” These results are similar to those of a previous study that reported that calf pain among patients in the early stage of DMD was related to prolonged daily toe walking, overuse syndrome (sprain and strain), and muscular fatigue owing to an increased demand on the gastrocnemius-soleus muscle complex [26]. This pain could be managed by tailoring the intensity of ambulation and exercise, maintaining the range of motion of the ankle.

Patients with DMD in the ENA group reported the knee as the most common site of pain, “transfer activity” as the most common aggravating factor, and “positional change” as the most common relieving factor. A potential hypothesis is that the movement of the knee joint may be induced by transfer activity, resulting in pain provocation. Even though joint contracture itself does not cause the pain, pain occurs when the joint and its capsule are pushed to their end range [27]. This hypothesis suggests the need to prevent or alleviate joint contracture even in the non-ambulatory stage.

Individuals in the LNA group reported the lumbosacral region as the most common site of pain, with other sites, including the chest-abdomen and buttocks (Fig. 2). They also indicated “sitting” as the most common pain aggravating factor, with “positional change” as the most frequently used pain-relieving method. Scoliosis and pelvic obliquity, which progress in the LNA period of the disease, are factors that negatively impact body alignment and posture and could result in the pain described by these individuals. Moreover, the progression of muscle weakness makes correcting the posture by themselves challenging, further causing or worsening the pain [27]. These explanations emphasize the importance of maintaining spine and pelvis alignment as well as developing an appropriate positioning program as the disease progresses to the LNA stage.

### Study limitations

The study has certain limitations, which need to be acknowledged for the interpretation of the results. First, all the participants were recruited from a single clinic at a tertiary hospital. The characteristics of pain may vary depending on how a particular clinic manages musculoskeletal conditions. Furthermore, since this was a cross-sectional study, we could not confirm the association between musculoskeletal problems (joint contractures and scoliosis) and pain; a longitudinal study should be conducted to investigate these associations.

## Conclusion

In patients with DMD, pain is a common symptom at all clinical stages. In particular, it is important to assess the pain of the patients in the LNA stage, since a higher frequency of pain, pain in more parts of the body, and more interference on mood by pain were noted in this group than in the other groups. Since each clinical stage has different characteristics, the prevention and coping strategies should be modulated accordingly.

## Supplementary Information

**Additional file 1: Supplementary Table 1.** Aggravating and relieving factors for pain.

## Data Availability

The datasets generated during and analyzed during the current study are not publicly available due to privacy and ethical restrictions but are available from the corresponding author on reasonable request.

## Abbreviations

Amb: ambulatory
BPI: Brief Pain Inventory
DMD: Duchenne muscular dystrophy
DMDSAT: DMD Functional Ability Self-Assessment Tool
ENA: Early non-ambulatory
KPD-Q: Korean version of the PainDETECT Questionnaire
LNA: Late non-ambulatory
NRS: Numeric rating scale

## References

[1] Hoffman EP, Brown RH, Kunkel LMJC (1987). Dystrophin: the protein product of the Duchenne muscular dystrophy locus. Cell.

[2] Broomfield J, Hill M, Guglieri M, Crowther M, Abrams KJN (2021). Life expectancy in Duchenne muscular dystrophy: reproduced individual patient data Meta-analysis. Neurology.

[3] Bushby K, Finkel R, Birnkrant DJ, Case LE, Clemens PR, Cripe L, Kaul A, Kinnett K, McDonald C, Pandya S (2010). Diagnosis and management of Duchenne muscular dystrophy, part 1: diagnosis, and pharmacological and psychosocial management. Lancet Neurol.

[4] Bushby K, Finkel R, Birnkrant DJ, Case LE, Clemens PR, Cripe L, Kaul A, Kinnett K, McDonald C, Pandya S (2010). Diagnosis and management of Duchenne muscular dystrophy, part 2: implementation of multidisciplinary care. Lancet Neurol.

[5] Birnkrant DJ, Bushby K, Bann CM, Alman BA, Apkon SD, Blackwell A, Case LE, Cripe L, Hadjiyannakis S, Olson AK (2018). Diagnosis and management of Duchenne muscular dystrophy, part 2: respiratory, cardiac, bone health, and orthopaedic management. Lancet Neurol.

[6] Guy-Coichard C, Nguyen DT, Delorme T, Boureau F (2008). Pain in hereditary neuromuscular disorders and myasthenia gravis: a national survey of frequency, characteristics, and impact. J Pain Symptom Manag.

[7] Pangalila RF, Van Den Bos GA, Bartels B, Bergen M, Stam HJ, Roebroeck ME (2015). Prevalence of fatigue, pain, and affective disorders in adults with Duchenne muscular dystrophy and their associations with quality of life. Arch Phys Med Rehabil.

[8] Tiffreau V, Viet G, Thévenon AJ (2006). Rehabilitation: pain and neuromuscular disease: the results of a survey. Am J Phys Med Rehabil.

[9] Hunt A, Carter B, Abbott J, Parker A, Spinty S, deGoede C (2016). Pain experience, expression and coping in boys and young men with Duchenne Muscular Dystrophy–a pilot study using mixed methods. Eur J Paediatr Neurol.

[10] Lager C, Kroksmark A-K (2015). Pain in adolescents with spinal muscular atrophy and Duchenne and Becker muscular dystrophy. Eur J Paediatr Neurol.

[11] Engel JM, Kartin D, Carter GT, Jensen MP, Jaffe KM (2009). Pain in youths with neuromuscular disease. Am J Hosp Palliat Med.

[12] Zebracki K, Drotar D (2008). Pain and activity limitations in children with Duchenne or Becker muscular dystrophy. Dev Med Child Neurol.

[13] Sox HC, Berwick DM, Berg AO, Frame PS, Fryback DG, Grimes DA, Lawrence RS, Wallace RB, Washington AE, Wilson MEJJ (1993). Screening for adolescent idiopathic scoliosis. JAMA.

[14] Brigham CR, Uehlein W, Uejo C, Dilbeck LJRD (2008). AMA guides sixth edition: perceptions, myths, and insights.

[15] Landfeldt E, Mayhew A, Eagle M, Lindgren P, Bell CF, Guglieri M, Straub V, Lochmüller H, Bushby KJND (2015). Development and psychometric analysis of the Duchenne muscular dystrophy functional ability self-assessment tool (DMDSAT). Neuromuscul Disord.

[16] Szabo SM, Audhya IF, Malone DC, Feeny D, Gooch KL. Characterizing health state utilities associated with Duchenne muscular dystrophy: a systematic review. Qual Life Res. 2020;29(3):593–605. 10.1007/s11136-019-02355-x PMC7028804 31811595

[17] Salerno DF, Franzblau A, Armstrong TJ, Werner RA, Becker MP (2001). Test-retest reliability of the upper extremity questionnaire among keyboard operators. Am J Ind Med.

[18] Palermo TM, Witherspoon D, Valenzuela D, Drotar DDJP (2004). Development and validation of the child activity limitations interview: a measure of pain-related functional impairment in school-age children and adolescents. Pain.

[19] Savedra MC, Tesler MD, Holzemer WL, Wilkie DJ, Ward JA (1989). Pain location: validity and reliability of body outline markings by hospitalized children and adolescents. Res Nurs Health.

[20] Margolis RB, Chibnall JT, Tait RCJP (1988). Test-retest reliability of the pain drawing instrument. Pain.

[21] Yun YH, Mendoza TR, Heo DS, Yoo T, Heo BY, Park H-A, Shin HC, Wang XS, Cleeland CSJO (2004). Development of a cancer pain assessment tool in Korea: a validation study of a Korean version of the brief pain inventory. Oncology.

[22] Freynhagen R, Baron R, Gockel U, Tölle TR (2006). pain DETECT: a new screening questionnaire to identify neuropathic components in patients with back pain. Curr Med Res Opin.

[23] Sung JK, Choi JH, Jeong J, Kim WJ, Lee DJ, Lee SC, Kim YC, Moon JY (2017). Korean version of the painDETECT questionnaire: a study for cultural adaptation and validation. Pain Pract.

[24] Choi Y-A, Chun S-M, Kim Y, Shin H-I (2018). Lower extremity joint contracture according to ambulatory status in children with Duchenne muscular dystrophy. BMC Musculoskelet Disord.

[25] McDonald CM, Abresch RT, Carter GT, Fowler WM, Johnson ER, Kilmer DD, Sigford BJ (1995). Profiles of neuromuscular diseases. Duchenne muscular dystrophy. Am J Phys Med Rehabil.

[26] Sutherland DH, Olshen R, Cooper L, Wyatt M, Leach J, Mubarak S, Schultz P (1981). The pathomechanics of gait in Duchenne muscular dystrophy. Dev Med Child Neurol.

[27] Engel JM, Kartin D, Jaffe KM (2005). Exploring chronic pain in youths with Duchenne muscular dystrophy: a model for pediatric neuromuscular disease. Phys Med Rehabil Clinics.

