# Supplementary material for: Pain characteristics among individuals with Duchenne muscular dystrophy according to their clinical stage
Source: BMC Musculoskelet Disord. 2022 Jun 4;23:536. doi: 10.1186/s12891-022-05504-5 (PMC9166361; doi:10.1186/s12891-022-05504-5)
Supplement: Supplementary file 1 — Additional file 1: Supplementary Table 1. Aggravating and relieving factors for pain. [file 12891_2022_5504_MOESM1_ESM.docx]

Supplementary Table 1. Aggravating and relieving factors for pain.

| Aggravating factors^a^ |  | Ambulatory,  N=17 | Early non-ambulatory,  N=11 | Late non-ambulatory,  N=21 | Total, N=49 |
| --- | --- | --- | --- | --- | --- |
|  | Ambulation | 14 (82.3%) | 0 | 0 | 14 (28.6%) |
|  | Stretch | 1 (5.9%) | 2 (18.2%) | 1 (4.8%) | 4 (8.2%) |
|  | Transfer activity | 0 | 5 (45.5%) | 3 (14.3%) | 8 (16.3%) |
|  | Excessive activity | 3 (17.6%) | 0 | 3 (14.3%) | 6 (12.2%) |
|  | Lying down | 2 (11.8%) | 1 (9.1%) | 4 (19.0%) | 7 (14.3%) |
|  | Sitting | 2 (11.8%) | 2 (18.2%) | 13 (61.9%) | 17 (34.7%) |
|  | Right after waking up | 0 | 1 (9.1%) | 1 (4.8%) | 2 (4.1%) |
|  | Poor general condition | 0 | 0 | 1 (4.8%) | 1 (2.0%) |
|  | When pressure is applied on a body part | 1 (5.9%) | 0 | 0 | 1 (2.0%) |
| Relieving factors^b^ |  | Ambulatory,  N=17 | Early non-ambulatory,  N=13 | Late non-ambulatory,  N=20 | Total, N=50 |
|  | Hot pack | 1 (5.9%) | 0 | 0 | 1 (2.0%) |
|  | Stretch | 3 (17.6%) | 1 (7.7%) | 1 (5.0%) | 5 (10.0%) |
|  | Analgesics | 1 (5.9%) | 0 | 2 (10.0%) | 3 (6.0%) |
|  | Massage | 5 (29.4%) | 4 (30.8%) | 3 (15.0%) | 12 (24.0%) |
|  | Positional change | 3 (17.6%) | 6 (46.2%) | 9 (45.0%) | 18 (36.0%) |
|  | Resting | 5 (29.4%) | 1 (7.7%) | 8 (40.0%) | 14 (28.0%) |
|  | Other methods^c^ | 0 | 0 | 3 (15.0%) | 3 (6.0%) |

^a, b^ When a participant gave more than one response, each was included in the analysis.

^c^ deep breathing, drinking hot water, adopting a specific posture
